# Desinfektionsmittel in der COVID-19-Pandemie: eine Herausforderung

**DOI:** 10.1007/s00103-021-03457-z

**Published:** 2021-12-08

**Authors:** Maren Eggers, Anna Baumann, Nils Lilienthal, Eike Steinmann, Jochen Steinmann, Nils-Olaf Hübner, Holger F. Rabenau, Viola Weinheimer, Ingeborg Schwebke

**Affiliations:** 1 Kommission für Virusdesinfektion, Deutsche Vereinigung zur Bekämpfung der Viruskrankheiten (DVV) e. V., Geschäftsstelle Kiel, Kiel, Deutschland; 2 Gesellschaft für Virologie (GfV) e. V., Geschäftsstelle Heidelberg, Heidelberg, Deutschland; 3 Labor Prof. G. Enders MVZ GbR, Rosenbergstraße 85, 70193 Stuttgart, Deutschland; 4 Bundesinstitut für Arzneimittel und Medizinprodukte (BfArM), Bonn, Deutschland; 5 Abteilung für Molekulare & Medizinische Virologie, Ruhr-Universität Bochum, Bochum, Deutschland; 6 Dr. Brill + Partner GmbH Institut für Hygiene und Mikrobiologie, Bremen, Deutschland; 7 Institut für Hygiene und Umweltmedizin, Universitätsmedizin Greifswald, Greifswald, Deutschland; 8 Institut für Medizinische Virologie, Universitätsklinikum Frankfurt am Main, Frankfurt am Main, Deutschland; 9 Bundesanstalt für Arbeitsschutz und Arbeitsmedizin (BAuA), Dortmund, Deutschland

**Keywords:** Coronavirus SARS-CoV-2, Desinfektion, Allgemeinverfügungen, Arzneimittel, Biozidprodukte, Coronavirus SARS-CoV-2, Disinfection, General ruling, Medicinal products, Biocidal products

## Abstract

Durch die COVID-19-Pandemie haben Desinfektionsmaßnahmen auch in Deutschland an Bedeutung gewonnen. Der erhöhte Bedarf an Desinfektionsmitteln zu Beginn der Pandemie erforderte es, vorübergehende rechtliche Regelungen zu treffen, um einerseits ausreichend Mittel für die notwendige Desinfektion im medizinischen Bereich und andererseits für den zusätzlichen Bedarf in der Bevölkerung zur Verfügung zu haben. Dazu wurden vom Bundesinstitut für Arzneimittel und Medizinprodukte (BfArM) und der Bundesanstalt für Arbeitsschutz und Arbeitsmedizin (BAuA) Allgemeinverfügungen erlassen, die in diesem Beitrag näher erläutert werden. Im Vordergrund stehen dabei die Maßnahmen für die hygienische Händedesinfektion. Aber auch weitere Anwendungen wie die Flächendesinfektion im Zusammenhang mit pandemischen Atemwegserkrankungen werden erörtert. Die Erfahrungen bei der Sicherstellung der Versorgung mit wirksamen und in der Anwendung sicheren Desinfektionsmitteln sollten für die Vorbereitung weiterer Pandemien genutzt werden.

## Abstract (en)

Disinfection measures have become more important as a result of the COVID-19 pandemic in Germany. The increased need for disinfectants at the beginning of the pandemic required temporary legal regulations in order to provide a sufficient quantity of products for the necessary disinfection in the medical sector on the one hand and for the additional demand in the population on the other. For this purpose, the Federal Institute for Drugs and Medical Devices (BfArM) and the Federal Institute for Occupational Safety and Health (BAuA) issued a general ruling, which is explained in more detail in this article. The focus was on measures for hygienic hand disinfection. However, other applications such as surface disinfection in relation to pandemic respiratory diseases are also addressed. The experience gained in ensuring the supply of disinfectants that are effective and safe to use should be used to prepare for further pandemics.

## Einleitung

Seit Beginn der durch SARS-CoV‑2 („severe acute respiratory syndrome coronavirus 2“) verursachten COVID-19-Pandemie haben sich weltweit ca. 262 Mio. Menschen infiziert; ca. 5,2 Mio. sind verstorben (Worldometer Corona 30.11.2021; [1]). Die schnelle pandemische Ausbreitung des Virus führte zur Frage nach geeigneten Schutzmaßnahmen im medizinischen und im öffentlichen Bereich. Damit rückte auch die Desinfektion in den Fokus des allgemeinen Interesses. In der Folge stieg die Nachfrage nach Desinfektionsmitteln nicht nur im medizinischen Bereich, sondern auch im öffentlichen und im privaten Umfeld stark an.

Gleichzeitig traten bestehende Wissenslücken und Fehleinschätzungen in Bezug auf adäquate und nichtadäquate Desinfektionsmaßnahmen hervor: Aktionen wie das Besprühen bzw. Benebeln ganzer Straßenzüge mit Desinfektionsmitteln prägten in mehreren Ländern das Bild der Coronavirusbekämpfung. In Deutschland konnte großflächiges Besprühen von Straßen verhindert werden, trotzdem stieg auch hierzulande die Nachfrage nach Desinfektionsmitteln auch in Bereichen, in denen diese bisher nicht oder kaum eingesetzt worden waren, stark an. Dies betraf zum einen Verbraucher aus Sorge, sich im öffentlichen Umfeld mit SARS-CoV‑2 zu infizieren – obwohl in verschiedenen Stellungnahmen darauf hingewiesen wurde, dass Händewaschen in der Regel ausreicht und eine Flächendesinfektion im Haushalt nicht erforderlich ist, so in den Stellungnahmen der Weltgesundheitsorganisation (WHO), des Robert Koch-Instituts (RKI), der Bundeszentrale für gesundheitliche Aufklärung (BZgA), der Desinfektionsmittelkommission der Deutschen Vereinigung zur Bekämpfung der Viruskrankheiten (DVV) und der Gesellschaft für Virologie (GfV; [2–4]).

Auch in vielen öffentlichen Bereichen, wie z. B. Supermärkten, Restaurants und Bahnhöfen, hoffte man durch Flächen- und Händedesinfektion die Pandemie eindämmen zu können. Vor allem nach dem ersten Lockdown setzten die örtlichen Behörden in Deutschland auf Hände- und Flächendesinfektion im öffentlichen Bereich. Allein für die Schulträger in Nordrhein-Westfalen wurden 20.000 l Desinfektionsmittel zum Abruf gestellt [5]. Durch diese zusätzliche Nachfrage nach Desinfektionsmitteln im Inland und bereits seit Anfang Januar durch das Ausland (z. B. China) kam es in Deutschland zur starken Verknappung etablierter Produkte.

**Figure 1:**
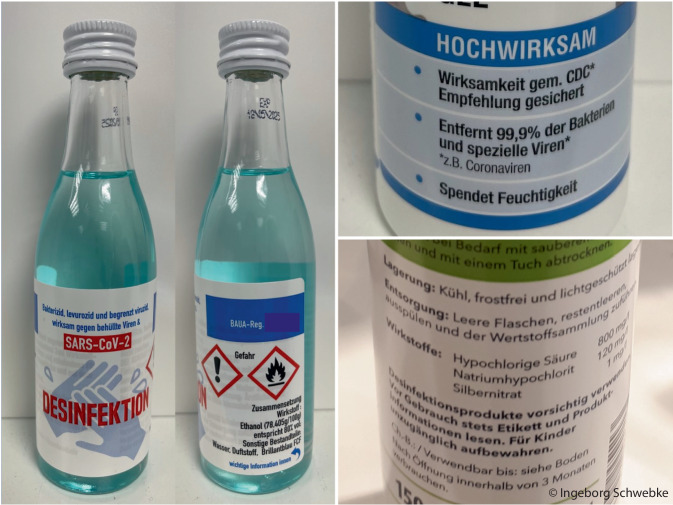

Eine Steigerung der Produktion war nur eingeschränkt möglich, da auch Verpackungen und Rohstoffe in der erforderlichen Qualität extrem knapp wurden. Die bisher verfügbaren Desinfektionsmittel, vor allem für die Händedesinfektion, waren zu diesem Zeitpunkt kaum noch erhältlich. Deshalb wurde intensiv nach Lösungen gesucht, um den Mangel möglichst schnell zu beseitigen und die Desinfektion vor allem im medizinischen Bereich im gewohnten Qualitäts- und Sicherheitsstandard durchführen zu können. Dazu war eine Kommunikationsstrategie notwendig, die den Anwender informieren, aber nicht verunsichern sollte. Währenddessen tauchten im Handel immer mehr neue und ungewöhnliche Produkte auf (Abb. 1). In Deutschland wurden in der Folge die Empfehlungen auf anerkannte Verfahren zur Desinfektion beschränkt.

Inwieweit die ergriffenen Maßnahmen zur Desinfektion ihrem Ziel gerecht wurden bzw. welche Bedeutung sie speziell bei SARS-CoV‑2 haben, lässt sich nach fast 2 Jahren Pandemie auf der Basis von Erfahrungen und wissenschaftlichen Erkenntnissen genauer einschätzen. Der vorliegende Beitrag beschreibt zunächst die Anforderungen an chemische Hände- und Flächendesinfektionsmittel und die rechtlichen Grundlagen für ihre Verkehrsfähigkeit. Im Folgenden werden die auf dieser Basis ergriffenen Maßnahmen und die speziellen Fragestellungen bei der Organisation der Desinfektionsmittelversorgung dargestellt. Dabei wird auch auf die Besonderheiten der Übergangsregelungen im Biozidrecht eingegangen. Des Weiteren wird die Bedeutung der Flächendesinfektion bei SARS-CoV‑2 erörtert. Abschließend werden die während der Pandemie gewonnenen Erkenntnisse bei der Organisation der Desinfektionsmittelversorgung dargestellt und Lehren gezogen, die dazu beitragen sollen, bei zukünftigen Pandemien bezüglich der Desinfektion besser vorbereitet zu sein.

## Anforderungen an chemische Desinfektionsmittel

Desinfektionsmittel müssen für den ausgelobten Wirkbereich (z. B. Bakterien, Hefen, Sporen, Viren) wirksam und für die jeweilige Anwendung weitgehend unbedenklich für Mensch und Umwelt sein. Der Nachweis der Wirksamkeit für den entsprechenden Wirkbereich muss durch standardisierte Prüfmethoden erbracht werden. Dazu existieren europäische Normen, deren Anwendung in der übergeordneten Norm DIN EN 14885 [6] geregelt ist. In Deutschland werden zum Teil darüber hinausgehende nationale Prüfvorschriften des Verbundes für Angewandte Hygiene e. V. (VAH), der DVV e. V. und GfV e. V. sowie des RKI angewendet. In jedem Fall sind orientierende Suspensionsversuche und praxisnahe Tests mit den für das jeweilige Wirkspektrum vorgegebenen Testorganismen durchzuführen. Nähere Erläuterungen zum erforderlichen Wirkspektrum und dem Nachweis der Wirksamkeit mit standardisierten Methoden für Händedesinfektionsmittel wurden im Epidemiologischen Bulletin 2020 veröffentlicht [7].

Aufgrund der biochemischen Struktur und der Erfahrungen mit SARS-CoV‑1 war bekannt, dass zur Desinfektion alkoholische Lösungen gut geeignet sind. Wie SARS-CoV‑1 gehört SARS-CoV‑2 zu den behüllten Viren, sodass zu seiner Inaktivierung der Wirkbereich „begrenzt viruzid“ ausreichend ist. Allgemeine Anforderungen an den Nachweis der Viruswirksamkeit hat der Arbeitskreis Viruzidie beim RKI formuliert [8].

Im medizinischen Bereich muss aber immer auch mit dem Vorhandensein weiterer Krankheitserreger gerechnet werden, sodass als Grundvoraussetzung bei Desinfektionsmitteln, neben einer Wirksamkeit gegen behüllte Viren (begrenzt viruzid), auch die Wirksamkeit gegen Bakterien und Hefen (Bakterizidie und Levurozidie) gegeben sein muss. Diese Anforderung gilt auch für den öffentlichen Bereich, wie z. B. Schulen, Feuerwehr oder Polizei, sowie für den privaten Bereich, wenn hier aus medizinischen Gründen eine Desinfektion erforderlich ist.

Desinfektionsmittel sollten jedoch nicht nur wirksam, sondern auch sicher in der Anwendung sein, was auch für die zu Pandemiebeginn dringend benötigten Produktalternativen galt.

## Rechtliche Grundlagen für die Verkehrsfähigkeit chemischer Desinfektionsmittel

**Figure 2:**
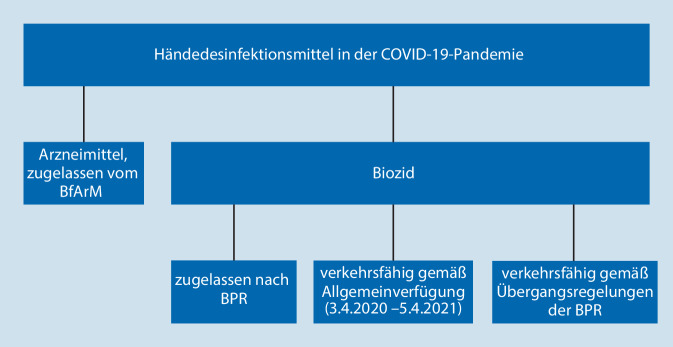

Händedesinfektionsmittel können rechtlich Biozidprodukte der Produktart 1 (PT 1, Produkte für die menschliche Hygiene) sein oder aufgrund von Bestandsschutz Arzneimittel (Abb. 2) sein. Flächendesinfektionsmittel gehören in der Regel zu den Biozidprodukten und hier zur Produktart 2 (PT 2). Unter Pandemiebedingungen waren vorübergehende juristische Regelungen für beide Rechtskreise, Biozidprodukte und Arzneimittel, erforderlich.

### Händedesinfektionsmittel als Arzneimittel oder Biozidprodukt

Neue Händedesinfektionsmittel werden als Biozidprodukte zugelassen, es sei denn, die beantragten Indikationen beinhalten zusätzlich zur hygienischen und chirurgischen Händedesinfektion auch die Verhütung von Krankheiten (z. B. Wundantiseptik; [9]). Die Zulassungsanforderungen für Händedesinfektionsmittel zum Beleg der Wirksamkeit ergeben sich allgemein aus der angestrebten Indikation – hygienische oder chirurgische Händedesinfektion – und dem beantragten Wirkspektrum.

Unterschiede in den Zulassungsanforderungen zwischen Händedesinfektionsmitteln als Arzneimittel und als Biozidprodukt wurden zuletzt ausführlich im Epidemiologischen Bulletin 17/2021 diskutiert [10]. Die Belege zum Wirksamkeitsnachweis stimmen jedoch weitestgehend überein.

Während der Pandemie wurde jedoch nicht nur die Neuzulassung von Händedesinfektionsmitteln wieder interessant, sondern auch die erweiterte Anwendung bereits zugelassener Händedesinfektionsmittel in der Verantwortung des Bundesinstituts für Arzneimittel und Medizinprodukte (BfArM). So ergaben sich in 6 Fällen Zulassungsänderungen zur Erweiterung des Wirkspektrums auf „begrenzt viruzid“ bzw. „begrenzt viruzid PLUS“, um die Wirksamkeit gegen behüllte Viren, die SARS-CoV‑2 einschließen, bzw. zusätzlich gegen die unbehüllten Adeno‑, Rota- und Noroviren, ausloben zu können.

### Flächendesinfektionsmittel als Biozidprodukt

Flächendesinfektionsmittel werden von den jeweiligen nationalen Zulassungsbehörden (in Deutschland: Bundesanstalt für Arbeitsschutz und Arbeitsmedizin [BAuA]) bzw. der Europäischen Chemikalienagentur (ECHA) auf der Basis der Anforderungen der Biozid-Verordnung (BPR; [11]) und zugehöriger Dokumente als Biozidprodukte zugelassen, Informationen hierzu siehe Website der BAuA [12].

### Ausnahmezulassungen nach Biozid-Verordnung (BPR)

Der Artikel 55 der BPR [11] regelt Ausnahmen des Zulassungsverfahrens. Das heißt, „eine zuständige Behörde“ kann „befristet für eine Dauer von höchstens 180 Tagen die Bereitstellung eines Biozidprodukts auf dem Markt oder die Verwendung eines Biozidprodukts, das nicht die in dieser Verordnung niedergelegten Voraussetzungen für die Erteilung einer Zulassung erfüllt, für eine beschränkte und kontrollierte Verwendung unter der Aufsicht der zuständigen Behörde gestatten, wenn dies aufgrund einer Gefahr für die öffentliche Gesundheit, die Tiergesundheit oder für die Umwelt notwendig ist, die mit anderen Mitteln nicht eingedämmt werden kann“. SARS-CoV‑2 stellt zwar keine besonders hohen Anforderungen an Desinfektionsmittel, aber die üblichen Produkte waren schnell nicht mehr in ausreichender Menge verfügbar. Deshalb trat dieser Artikel im humanmedizinischen Bereich erstmals in Kraft, damit auch nicht regulär zugelassene Produkte sicher angewendet werden konnten.

**Figure 3:**
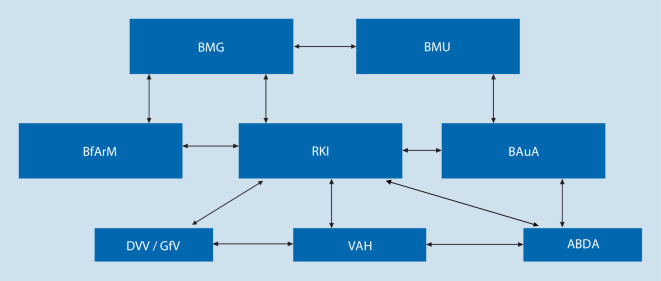

Einzelheiten für das Vorgehen nach Artikel 55 BPR sind für Deutschland in § 12 Chemikaliengesetz (ChemG; [13]) festgelegt. Die BAuA als reguläre Zulassungsbehörde verfügte über jahrelange Erfahrung in Zulassungsverfahren; hier jedoch mussten schnell Produkte für die „Ausnahmezulassung“ gefunden werden, die einerseits wirksam und andererseits auch sicher in der Anwendung sind. In enger Zusammenarbeit mit dem RKI und den beiden zuständigen Ministerien – Ministerium für Umwelt, Naturschutz und nukleare Sicherheit (BMU) sowie dem Bundesministerium für Gesundheit (BMG) – entstanden Allgemeinverfügungen für die hygienische Händedesinfektion und die Flächendesinfektion ([14, 15]; Abb. 3).

## Wege aus der Produktknappheit – Allgemeinverfügungen und Standardzulassung

Um der Dringlichkeit des pandemiegeschuldet erhöhten Bedarfs an Händedesinfektionsmitteln Rechnung zu tragen, wurden zeitlich befristete Abweichungen von den allgemeinen Standards der Zulassung für alkoholhaltige Arzneimittel und Biozidprodukte zur hygienischen Händedesinfektion erlaubt. Hierzu erließen das BfArM und die BAuA im März 2020 Allgemeinverfügungen, um die Verfügbarkeit von Händedesinfektionsmitteln unter Beibehaltung eines hohen Mindeststandards aufrechtzuerhalten [14, 16].

### Allgemeinverfügung des BfArM für Händedesinfektionsmittel

Die Allgemeinverfügung des BfArM [16] erlaubte beispielsweise – vorbehaltlich der Gewährleistung gleicher Unbedenklichkeit und Wirksamkeit gegen Viren – Freiheiten bei der Wahl des Wirkstofflieferanten, der Packmittel und deren Farben sowie die Substitution nicht wirksamkeitsrelevanter Hilfsstoffe. Zusätzlich wurde die Spezifikation zur Sporenfreiheit in Arzneimitteln ausgesetzt, um die Freigabe zu beschleunigen. Durch diese Allgemeinverfügung konnte bei mindestens 2 Firmen die Lieferfähigkeit essenzieller Produkte zur Händedesinfektion aufrechterhalten werden. Die Allgemeinverfügung des BfArM bezog sich jedoch ausdrücklich nur auf Desinfektionsmittel zur hygienischen Händedesinfektion. Für die chirurgische Händedesinfektion wurden keine vorrübergehenden Abweichungen ermöglicht, um den hohen Standards der Krankenhaushygiene und dem Schutz der Patienten vor postoperativen Infektionen gerecht zu werden.

### Allgemeinverfügung der BAuA für Händedesinfektionsmittel

Die Allgemeinverfügung der BAuA [14] beinhaltete die Herstellung und Verwendung 2‑propanolhaltiger und ethanolhaltiger Händedesinfektionsmittel nach vorgegebenen Rezepturen.

Der Versorgungsengpass bei Desinfektionsmitteln führte auch schnell zu der Frage einer möglichen Eigenherstellung und den damit verbundenen Fragen: Welche Rezepturen sind geeignet und was ist bei der Herstellung zu beachten? Erste Überlegungen führten zu den WHO-Formulierungen [17], die vorrangig für Länder gedacht sind, die keine eigene Produktion besitzen und die die relativ teuren Produkte der westlichen Welt nicht erwerben können. Diese Fragen wurden bereits im März 2020 über die Veröffentlichung der Desinfektionsmittelkommission der DVV e. V. und GfV e. V. [2] parallel zur ersten Allgemeinverfügung der BAuA adressiert.

Die wichtigsten Wirkstoffe der etablierten Händedesinfektionsmittel sind Ethanol, Isopropanol (2-Propanol) und 1‑Propanol. Nachdem konfektionierte Produkte mit diesen Wirkstoffen nicht mehr zur Verfügung standen, wurden für die Allgemeinverfügung die beiden WHO-Formulierungen I und II [17] auf der Basis von Ethanol bzw. Isopropanol herangezogen, da zu ihrer Wirksamkeit – auch gegen Viren – eine Vielzahl von Publikationen vorlag [18–20]. In nachfolgenden wissenschaftlichen Untersuchungen bestätigte sich ihre Wirksamkeit auch gegenüber dem behüllten SARS-CoV‑2 [21].

Neben den in Deutschland vor der Pandemie nicht verwendeten WHO-Formulierungen existieren im Arzneimittelbereich die Standardzulassungen verschiedener alkoholischer Lösungen, für deren Wirksamkeit auch gegen behüllte Viren ebenfalls wissenschaftliche Untersuchungen vorlagen und die in die Desinfektionsmittelliste des RKI aufgenommen waren [22–25]. Sie haben z. T. zusätzlich den Anwendungsbereich der Hautdesinfektion, für den ebenfalls die Mittel knapp wurden.

Über die korrekte Herstellung der Lösungen herrschten allerdings vor allem bei in der Desinfektionsmittelproduktion unerfahrenen Firmen viele Unklarheiten. Lösungen z. B. mit einem vorgegebenen Ethanolgehalt herzustellen ist keineswegs trivial, da substanztypische Eigenschaften wie die Volumenkontraktion bei Verdünnung und die Dichte zu beachten sind. Mit Unterstützung der Bundesvereinigung Deutscher Apothekerverbände e. V. (ABDA) gelang es, eine nachvollziehbare Herstellungsanweisung zu erstellen, die dann in der endgültigen Fassung der Allgemeinverfügung veröffentlicht wurde [14]. Eine solche detaillierte Vorschrift wurde notwendig, da aus Kapazitätsgründen nun nicht nur die erfahrenen Hersteller, sondern auch weitere Firmen der pharmazeutischen und chemischen Industrie sowie juristische Personen des öffentlichen Rechts die Möglichkeit erhielten, die Lösungen gemäß Allgemeinverfügung herzustellen.

Hintergründe zur Wirksamkeit und Unbedenklichkeit der verschiedenen Rezepturen wurden detailliert in einem Artikel des RKI zusammen mit der DVV und GfV und weiteren Experten zum Tag der Händehygiene 2020 dargestellt [7]. Der VAH veröffentlichte zeitgleich eine weitere Mitteilung [26], die beinhaltete, dass nur bei Ethanol-Wasser-Gemischen ab 80 % (v/v)^1^ und 2‑Propanol-Wasser-Gemischen ab 75 % (v/v) auch von einer ausreichenden bakteriziden Wirksamkeit nach 0,5 min Einwirkzeit ausgegangen werden kann. Für die Anwendung von Hautantiseptika empfahl die VAH-Desinfektionsmittelkommission vorzugsweise die alkoholischen Lösungen der Standardzulassung zu nutzen, da bei sachgerechter Herstellung gemäß den entsprechenden Monografien der Standardzulassung des BfArM auch eine Sporenfreiheit der Lösungen gegeben wäre. Bei Produkten, die als Biozide oder gemäß der von der BAuA erlassenen Allgemeinverfügung vermarktet wurden, muss dies nicht der Fall sein.

### Standardzulassungen für alkoholische Lösungen

Standardzulassungen stellen bestimmte Fertigarzneimittel gemäß § 36 Arzneimittelgesetz (AMG) von der Zulassungspflicht frei. Voraussetzung hierfür ist, dass keine Gefährdung von Menschen und Tieren zu befürchten ist. Sie basieren auf Monografien, die das BMG in Kraft setzt. Sie werden im Bundesanzeiger veröffentlicht und dürfen nur den jeweiligen Alkohol (auch mit Butan-2-on vergällt) und Wasser enthalten. Ihre Herstellung unter Normalbedingungen bedarf der Anzeige beim BfArM und der zuständigen Landesbehörde.

Diese Ausnahme von der Zulassungspflicht über die Standardzulassung wurde in den letzten Jahren teilweise kritisch gesehen, da keine tiefergehende Prüfung der Anträge auf Standardzulassung erfolgte und diese Regelung eine nationale Besonderheit darstellt, die im harmonisierten europäischen Arzneimittelrecht nicht vorkommt. Sie erlaubte aber während der Pandemie Apotheken, pharmazeutischen und chemischen Unternehmen sowie juristischen Personen des öffentlichen Rechts relativ unproblematisch Händedesinfektionsmittel herzustellen. So gingen nach Jahren ohne neue Anzeigen ab Pandemiebeginn Mitte März 2020 insgesamt 35 neue Anzeigen von Standardzulassungen alkoholischer Lösungen beim BfArM ein. Die Hürden für die Herstellung wurden durch die Allgemeinverfügung nochmals herabgesetzt.

Eine der Lehren aus der Pandemie sollte daher sein, über den Fortbestand der Standardzulassung für diese Alkohole nachzudenken, um in der Zukunft flexibler auf Lieferengpässe reagieren zu können.

Mit zunehmender Verbesserung der Versorgungsstruktur und Verfügbarkeit von Desinfektionsmitteln liefen die Allgemeinverfügung des BfArM [16] zum 30.09.2020 und die Allgemeinverfügungen der BAuA [14] zum 05.04.2021 aus.

### Mehr Freiheiten bei der pharmazeutischen Qualität von Händedesinfektionsmitteln

Speziell bei Händedesinfektionsmitteln ist die Qualität der Rohstoffe besonders wichtig, da hier ein Produkt regelmäßig auf lebendes Gewebe – die menschliche Haut – aufgebracht wird. Bereits die erste Version der Allgemeinverfügung der BAuA berücksichtigte diesen Aspekt, indem die Qualität der Mittel so definiert wurde, dass weder CMR (kanzerogene, mutagene, reproduktionstoxische) noch sensibilisierende Bestandteile über einem zulässigen Grenzwert (≤0,1 %) enthalten sein durften.

Im Gegensatz zu regulär zugelassenen Händedesinfektionsmitteln, für die die Art der Verpackung vorgeschrieben ist, wurde durch die Allgemeinverfügungen auch die Verpackung in riesigen Gebinden – bis 1000 l – möglich. Doch wie kann aus solchen Gebinden eine Abfüllung in handliche Gefäße unter hygienischen Bedingungen erfolgen? Da es im Biozidrecht keine Vorgaben zur mikrobiologischen Reinheit gibt, blieb das eine rechtlich ungelöste Frage, die geeignete Antworten durch die Anwender und Überwachungsbehörden forderte.

Außerdem waren die wichtigsten Wirkstoffe für Händedesinfektionsmittel – Ethanol und Isopropanol – nicht in ausreichender Menge in der erforderlichen pharmazeutischen Qualität verfügbar. Um auch weitere Quellen für alkoholische Wirkstoffe nutzen zu können, wurde der Artikel 95 der BPR [11] ausgesetzt. Dieser Artikel verweist auf eine Liste der Wirkstoffhersteller, die die Dossiers für das jeweilige Wirkstoffgenehmigungsverfahren erstellt haben und nutzen dürfen. Daher zeigte sich auch unter Pandemiebedingungen die essenzielle Bedeutung des Wirkstoffes Ethanol, da hierfür notfalls weitere Quellen verfügbar wären und Ethanol zur Inaktivierung von Viren eine große Bedeutung hat [27].

Es ergab sich allerdings ein Problem: So wollten z. B. Spirituosen- und Bierhersteller Ethanol aus ihrer eigenen Herstellung zur Verfügung stellen. Ein nicht näher charakterisierter Rohethanol kann aber nicht ohne Weiteres für die Anwendung auf der Haut freigegeben werden, da u. a. Fuselstoffe die Haut reizen können. Aber nicht nur die Reinheit, sondern auch der Ethanolgehalt solcher Rohfraktionen war nicht in jedem Fall geeignet.

Verunreinigungen von Produkten mit Methanol, die in den USA zu umfangreichen Warnhinweisen und Rückrufen geführt hatten [28, 29], wurden auch in Deutschland festgestellt. So mussten 2 Einzelhandelsketten ein Produkt (Handgel) zurückrufen, dass 65,3 g beziehungsweise 68,7 g Methanol pro 100 g Produkt enthielt^2^ [30]. Das Produkt stammte von einem Großhändler für alkoholische Getränke, Tabakwaren, Kosmetik und Feinkost, der keine Genehmigung zum Verkauf des Handgels hatte.

### Allgemeinverfügung für Flächendesinfektionsmittel

Neben der Allgemeinverfügung für Händedesinfektionsmittel, konnte vom 02.04.2020 bis 30.09.2020 eine Allgemeinverfügung der BAuA zum Inverkehrbringen von Flächendesinfektionsmitteln genutzt werden [15]. Sie enthielt 80 % (v/v) Ethanol-, 0,5 % (w/w^3^) Natriumhypochlorit- und 2,5 % (w/w) Chloramin-T-Lösung mit entsprechenden Qualitätsvorgaben.

## Übergangsregelungen im Biozidrecht

Neben diesen nun für den „Notfall“ per Allgemeinverfügung zugelassenen Produkten gibt es aufgrund der Übergangsregelungen für Biozidprodukte mit sogenannten Altwirkstoffen in Deutschland auch viele weitere Produkte mit anderen Wirkstoffen, die derzeit noch ohne Zulassung verkehrsfähig sind. Diese werden vom Inverkehrbringer lediglich elektronisch bei der BAuA gemäß der Meldeverordnung [31] registriert. Eine behördliche Prüfung hinsichtlich ihrer Wirksamkeit, Unbedenklichkeit und Qualität ist für derartige Meldungen gesetzlich nicht vorgeschrieben. Man erkennt sie an der Registriernummer mit der Struktur „N-xxxxx“.

Die gemeldeten Biozidprodukte mit Altwirkstoffen, also Produkte, die ausschließlich Wirkstoffe enthalten, die entsprechend der Review-Verordnung (Verordnung (EU) Nr. 1062/2014; [32]) bewertet wurden bzw. bewertet werden, können in Deutschland bis zur (Nicht‑)Genehmigung der enthaltenen Wirkstoffe ohne Zulassung vermarktet und verwendet werden. Bis spätestens zum Zeitpunkt der Genehmigung des Wirkstoffs muss jedoch ein vollständiger Antrag auf Zulassung gestellt werden, um die Verkehrsfähigkeit zu erhalten. Enthält ein Produkt mehrere Altwirkstoffe, gilt als Frist der Zeitpunkt des letzten zu genehmigenden Wirkstoffs (vorausgesetzt, es kam nicht zu einer Nichtgenehmigung eines der Wirkstoffe).

### Spielräume der Übergangsregelungen für neue oder (wieder-)entdeckte Produkte

Einige „neue“ Produkte bzw. bereits auf dem Markt befindliche Produkte mit Aktivchlor als Wirkstoff wurden während der Pandemie (wieder-)entdeckt und teils aggressiv beworben (Abb. 1).

Die oben genannten Übergangsregelungen wurden von Herstellern, deren Produkte bei Desinfektionsmaßnahmen im medizinischen Bereich in der Regel nicht zur ersten Wahl gehörten, verstärkt genutzt. Aufgrund des Mangels an geeigneten alkoholischen Rohstoffen im Frühjahr 2020 sprangen Hersteller von Chlorprodukten in diese Lücke und bewarben ihre Produkte für die Bekämpfung der Pandemie. Diese Produkte enthielten vorrangig Aktivchlor, das aus Natriumhypochlorit, hypochloriger Säure oder Salzlösungen hergestellt ist. Die Mehrzahl der Produkte war durch Übergangsregelungen verkehrsfähig und noch nicht bewertet.

Für Produkte zur Händedesinfektion (d. h. PT 1) mit dem Wirkstoff Natriumhypochlorit hätte bereits zum 01.01.2019 ein Zulassungsantrag gestellt werden müssen. Einige Firmen fanden allerdings Wege, den erforderlichen Zulassungsantrag zu umgehen. Dazu setzten sie z. B. Ethanol in minimaler Menge zu, da dieser Wirkstoff noch nicht genehmigt ist, oder meldeten das entsprechende Produkt zusätzlich für eine Produktart an, für die das Aktivchlor noch nicht genehmigt war, wie z. B. PT 11 – Schutzmittel für Flüssigkeiten in Kühl- und Verfahrenssystemen. Damit war es möglich, solche Aktivchlor abspaltenden Produkte ohne Zulassung, d. h. ohne eine Bewertung, sogar für die Händedesinfektion zu vermarkten.

Aufgrund des allgemeinen Desinfektionsmittelmangels wurden diese Produkte nun auch in größerem Umfang genutzt. Dadurch offenbarten sich aber auch ihre Schwachpunkte wie relativ lange Einwirkzeiten oder die häufig geringe Haltbarkeit infolge der Instabilität der Wirkstoffe. Überwachungsbehörden berichteten dem RKI zudem mehrfach von stark schwankenden Wirkstoffgehalten. Somit erfordert die Anwendung solcher Produkte eine regelmäßige Überprüfung des Wirkstoffgehalts. Zur Information der Anwender hatte die Desinfektionsmittelkommission des VAH zwei diesbezügliche Mitteilungen veröffentlicht [33, 34]. Aufgrund der fehlenden Erfahrung mit häufigen Anwendungen wird darin von der Verwendung chlorhaltiger Produkte für die Händedesinfektion abgeraten, solange diese nicht zugelassen sind, und eine Listung wird von der Zulassung abhängig gemacht.

Aus Sicht der Arzneimittelzulassung sind diese Produkte prinzipiell zur Händedesinfektion geeignet. Allerdings ist von einer allgemeinen Verwendung chlorhaltiger Produkte zur Händedesinfektion abzuraten, da Natriumhypochloritlösungen in der Regel pH-Werte deutlich im alkalischen Bereich aufweisen, während der pH-Wert der Haut im schwach sauren Bereich liegt. Hautirritationen können bei einer wiederholten Verwendung grundsätzlich nicht ausgeschlossen werden. Für die wiederholte hygienische Händedesinfektion werden alkoholbasierte Händedesinfektionsmittel empfohlen [35]. Dennoch wird auch hier eine Intensivierung der Hautpflegemaßnahmen nahegelegt. Aus den oben genannten Gründen des Hautschutzes wird nur die Verwendung ausschließlich für die Händedesinfektion zugelassener Produkte angeraten.

Die Zuordnung des richtigen Wirkstoffes bei Produkten, die Aktivchlor entwickeln, ist kompliziert. Deshalb hat die BAuA im Helpdesk hierzu eine Information (FAQ Nr. 0598) veröffentlicht [36]. Inzwischen ist die Bewertung weiterer Aktivchlor abspaltender Wirkstoffe abgeschlossen und sie wurden als „genehmigte“ Wirkstoffe in die Unionsliste [37] für PT 1 und PT 2 (z. B. als Flächendesinfektionsmittel) aufgenommen. Damit ist auch ein Ende der Nutzung von Übergangsregelungen vorgegeben. Im Rahmen der Bewertung im Zulassungsverfahren wird sich zeigen, unter welchen Gegebenheiten Aktivchlor abspaltende Verbindungen sicher zur Desinfektion eingesetzt werden können.

## Flächendesinfektion bei SARS-CoV-2

Bei zahlreichen Virusinfektionen erfolgt die Übertragung humanpathogener Viren nicht nur über Tröpfchen oder aerogen, sondern auch über kontaminierte Flächen. Dabei ist im Allgemeinen die Stabilität behüllter, humanpathogener Viren wie SARS-CoV‑2 durch den Lipidanteil in ihrer Membran geringer als die unbehüllter Viren (z. B. Noroviren). Wichtige Faktoren für die Stabilität der Viren in der Umwelt sind ferner mögliche Eiweiß- und Blutzusätze, die Temperatur, die Luftfeuchtigkeit und die Art bzw. das Material der Fläche. Nach der Übersicht von Kramer et al. liegt die Zeit bis zur Inaktivierung auf der Fläche bei behüllten Viren aus dem Bereich des Respirationstraktes und dem der blutübertragenen Viren (HIV, Hepatitisviren) im Bereich von wenigen Tagen, während für unbehüllte Viren, die überwiegend aus dem Gastrointestinaltrakt stammen, sogar Wochen und Monate angegeben werden [38, 39].

Zu Beginn und auch im weiteren Verlauf der COVID-19-Pandemie ist zurecht dann auch immer wieder hinterfragt worden, wo, wie und in welchem Umfang eine Kontamination von patientennahen Flächen durch Personen mit positivem SARS-CoV-2-Nachweis im Nasen‑/Rachenabstrich erfolgen kann. Deshalb sind bereits zu Beginn der Pandemie Untersuchungen zum Nachweis und zur Stabilität von SARS-CoV‑2 auf unterschiedlichen Flächen erfolgt [40, 41]. Viele der erhobenen Daten zur Stabilität auf der Fläche basieren jedoch nicht auf der Detektion von infektiösen Viruspartikeln, nachgewiesen in der Zellkultur, sondern von genetischem Material, welches in diesen Fällen mit der Polymerasekettenreaktion (PCR) detektiert wird. Eine Studie aus Wuhan zeigte beispielsweise, dass mit der PCR in Patientenzimmern das genetische Material bis zu 28 Tage nachgewiesen werden konnte [42]. Ein Virusnachweis in der Zellkultur ist hier nicht versucht worden. In einer anderen Studie wurde in 52,3 % der Proben in einem Londoner Krankenhaus genetisches Material detektiert [43]. Ein direkter Nachweis infektiöser Viren gelang hier nicht. Eine Studie aus einem Krankenhaus in Singapur ergab ein unklares Bild der Viruskontamination mit negativen und positiven Befunden, wobei die Nachweise mit der PCR hauptsächlich im Badezimmer erfolgreich waren [44]. Diese und viele weitere Studien zeigen das Ausmaß einer möglichen Kontamination im medizinischen Bereich. Der Versuch einer Virusisolierung fehlt aber häufig bzw. zeigte ein negatives Ergebnis. Deshalb wurde in der Folge intensiv diskutiert, welche Bedeutung positive PCR-Befunde für die Notwendigkeit einer Flächendesinfektion im medizinischen Bereich überhaupt haben [45].

Ein wichtiges Hilfsmittel für die Bewertung PCR-positiver Proben könnte der sogenannte Ct(Cycle-Threshold)-Wert sein, mit dem beschrieben wird, wie viele Zyklen in der PCR für die Generierung eines positiven Resultates erforderlich sind. Aber auch diese ergänzende Angabe ersetzt nicht den fehlenden Virusnachweis. Im nichtmedizinischen Bereich wird die durchgeführte Flächendesinfektion kritisch gesehen, wobei auch hier der fehlende Virusnachweis mit der Zellkultur als wichtiges Kriterium für die vorgenommene Einschätzung angesehen wird [46].

Daher weisen die US-amerikanischen Centers for Disease Control and Prevention (CDC) in einer im April 2021 aktualisierten Stellungnahme [47] darauf hin, dass durch Kontakt mit Oberflächen SARS-CoV‑2 übertragen werden könnte. Die Übertragung über Oberflächen stellt – basierend auf den verfügbaren epidemiologischen Daten und Studien – jedoch nicht den Hauptübertragungsweg von SARS-CoV‑2 dar und das Risiko wird als gering eingestuft. Klar ist, dass SARS-CoV‑2 primär durch Aerosole oder Tröpfchen, die auf die Atemwegsschleimhäute gelangen, übertragen wird.

Daher wäre es in den meisten Situationen im nichtmedizinischen Bereich ausreichend, die Oberflächen mit Seife oder Reinigungsmittel zu reinigen und nicht zu desinfizieren.

In Situationen, in denen innerhalb der letzten 24 h ein Verdachtsfall oder ein bestätigter Fall von COVID-19 in Innenräumen aufgetreten ist, ist das Vorhandensein infektiöser Viren auf Oberflächen wahrscheinlicher. Die Desinfektion von häufig berührten Oberflächen sollte daher in Betracht gezogen werden [48].

Wie bei den Stabilitätsdaten auf der Fläche sind viele Rückschlüsse zur chemischen Inaktivierung des SARS-CoV‑2 von anderen Mitgliedern der großen Coronavirusfamilie abgeleitet, wobei sicherlich von einem weitestgehend identischen Verhalten der einzelnen Familienmitglieder der *Coronaviridae* gegenüber chemischen Flächendesinfektionsmitteln ausgegangen werden kann.

Neuere gezielte Untersuchungen von Handelspräparaten zur Flächendesinfektion mit dem SARS-CoV‑2 sind in Deutschland und Europa nur im Einzelfall erforderlich. In den vorhandenen Prüfnormen [6] werden in der Regel einzelne apathogene und möglichst gut zu vermehrende Vertreter mit hohen Titern in der Zellkultur als Prüfviren im quantitativen Suspensionsversuch und in praxisnahen Versuchen unter Belastung eingesetzt. Bekanntlich fungiert bei der Prüfung der Viruzide der chemischen Flächendesinfektionsmittel das Vacciniavirus als Prüfvirus für alle behüllte Viren (begrenzt viruzid) und erlaubt so auch eine sichere Aussage zur Wirksamkeit gegenüber dem SARS-CoV‑2 [49, 50].

Mit dieser Vorgehensweise bei der Prüfung von chemischen Desinfektionsmitteln können so geprüfte und zertifizierte Flächendesinfektionsmittel auch in Zeiten von COVID-19 zielgerecht zur Inaktivierung des SARS-CoV‑2 eingesetzt werden. Geprüfte Flächendesinfektionsmittel mit einem erweiterten Wirkbereich wie „begrenzt viruzid PLUS“ und „viruzid“ können ebenfalls erfolgreich bei der Inaktivierung des SARS-CoV‑2 Verwendung finden.

## Lehren aus der Pandemie

Die Erfahrungen aus knapp 2 Jahren COVID-19-Pandemie zeigen, dass mit Artikel 55 der BPR in Europa eine tragfähige rechtliche Vorgabe existiert, um im Bedarfsfall schnell zusätzliche Produkte zur Desinfektion verfügbar zu machen. Die größte Herausforderung bestand dabei darin sicherzustellen, dass trotzdem nur wirksame und verträgliche Produkte auf den Markt gelangen.

Hier zeigten sich die Grenzen der aktuellen Gesetzgebung: Weder das europäische noch das deutsche Biozidrecht enthielten Instrumente, um zu verhindern, dass unter Pandemiebedingungen ungeprüfte Produkte massenhaft vermarktet wurden. Nachbesserungen hinsichtlich der Verkehrsfähigkeit insbesondere für Händedesinfektionsmittel als Produkte, die unmittelbar auf der menschlichen Haut angewendet werden, erscheinen dringend geboten.

In jedem Fall müssen auch Produkte für eine „Notfallzulassung“ wirksam und sicher bzw. unbedenklich in der Anwendung sein. Die Prüfung der Wirksamkeit und Sicherheit muss schon vor einer Pandemie oder einer anderen „Gefahr für die öffentliche Gesundheit“ erfolgen, da sie einen längeren Zeitraum in Anspruch nimmt. Somit ist es erforderlich, Produkte für ein breites Wirkspektrum vorzuhalten, um im Bedarfsfall schnell reagieren zu können, wozu über die Zulassung der Biozidprodukte hinaus Desinfektionsmittellisten, wie z. B. die des RKI oder des VAH, beitragen können.

Eine weitere Lehre aus der Pandemie ist die Verbesserung der Vorsorge (Preparedness) zur Verhinderung von Mangelsituationen: Eine gravierende Desinfektionsmittelknappheit war vor der Pandemie nicht im Fokus der Vorbereitungsmaßnahmen. Zukünftig sollte daher die Sicherstellung der Versorgungssicherheit mit Desinfektionsmitteln und Antiseptika für den medizinischen Bereich in Zeiten stark erhöhter Nachfrage und des Mangels an Rohstoffen etc. Teil der Vorbereitung sein. Zusätzlich sollte über den Fortbestand der Standardzulassung für alkoholische Lösungen nachgedacht werden, um flexibler auf Lieferengpässe reagieren zu können.

Die Pandemie hat zudem deutlich gemacht, dass eine intensive Kooperation der mit Desinfektionsmitteln befassten Bundesbehörden und Ministerien für sachdienliche Lösungen unumgänglich ist. Es wäre wünschenswert, diese Zusammenarbeit z. B. in einem Arbeitskreis dauerhaft zu regeln, um bei Produkten, die mit medizinischer Zweckbestimmung eingesetzt werden sollen, infektionshygienische und anwendungsrelevante Aspekte besser berücksichtigen zu können. Hierbei sollte zudem die in unabhängigen Expertengremien wie der DVV, dem VAH oder der Deutschen Gesellschaft für Krankenhaushygiene (DGKH) vorhandene Kompetenz eingebunden werden.

Derzeit ist die Versorgung mit Desinfektionsmitteln wieder gesichert und Ausweichprodukte sind entbehrlich. Das ist ein glücklicher Umstand und nicht selbstverständlich. Auch ist zu bedenken, dass es im Fall von SARS-CoV‑2 als behülltem Virus recht unproblematisch war, geeignete Produkte zur sicheren Inaktivierung zu identifizieren. Bei stabileren Erregern und solchen, bei denen durch eine Händewaschung und Flächenreinigung keine ausreichende Wirkung zu erzielen ist, dürfte es schwieriger sein, geeignete Produkte zu identifizieren als auch bereitzustellen, da die Nachfrage nochmals wesentlich größer sein dürfte.

Daher ist die COVID-19-Pandemie auch als ein Test für die Krisenfestigkeit der Versorgung mit Desinfektionsmitteln zu sehen. Dabei sind die Stärken, aber auch die Defizite deutlich geworden. Der Blick in die Geschichte zeigt, dass Pandemien regelmäßige Ereignisse sind. Ob in der nächsten Pandemie eine sichere Versorgung mit Desinfektionsmitteln, Masken, persönlicher Schutzausrüstung etc. gegeben ist, wird dagegen maßgeblich davon abhängen, ob die Lehren aus dieser Pandemie berücksichtigt und die Defizite behoben werden.

Bei alledem sollten auch die Kommunikation und Transparenz zur Notwendigkeit und Sinnhaftigkeit von Desinfektionsmaßnahmen nicht unterschätzt werden. So ist die grundsätzliche Anwendung von Hände- und Flächendesinfektionsmitteln im Rahmen eines überwiegend respiratorisch übertragenen Erregers kritisch bzw. differenziert zu betrachten. Neben der sinnvollen und unzweifelhaften Anwendung von Desinfektionsmitteln im medizinischen Bereich hat die Desinfektionsmittelbereitstellung und Nutzung z. B. in Kaufhäusern, Restaurants oder im Privatbereich eher einen stark eingeschränkten medizinisch-biologischen Nutzen, während die psychologische Komponente dieser Maßnahme (im Sinne „jeder kann selbst etwas tun und ist nicht nur ausgeliefert“) nicht unterschätzt werden darf. Nichtsdestotrotz sollte gerade für den Privatbereich bzw. überall dort, wo möglich, betont werden, dass man mit einfachem Händewaschen oftmals eine Übertragung effizient bekämpfen kann.

## Footnotes

1 (v/v) Konzentrationsangabe in Volumenprozent.

2 www.produktwarnung.eu: „Ein Teil der Produkte enthält eine hohe Menge an Methanol (65,3 g bzw. 68,7 g pro 100 g), das eine toxische Wirkung hat. Der Hersteller verfügt nicht über die Erlaubnis, die Produkte in Verkehr zu bringen und hat die Artikel nicht mit den notwendigen kindergesicherten Verschlüssen versehen. Gesundheitsschädliche Auswirkungen können nicht ausgeschlossen werden, insbesondere bei Verschlucken, Hautkontakt oder Einatmen des Inhalts. Von einem Gebrauch wird daher abgeraten“.

3 (w/w) Konzentrationsangabe in Massenprozent.
